# Microcystin Contamination and Toxicity: Implications for Agriculture and Public Health

**DOI:** 10.3390/toxins14050350

**Published:** 2022-05-17

**Authors:** Rajesh Melaram, Amanda R. Newton, Jennifer Chafin

**Affiliations:** Department of Science, Eastern Florida State College, Melbourne, FL 32935, USA; newtona@easternflorida.edu (A.R.N.); chafin.jennifer@titans.easternflorida.edu (J.C.)

**Keywords:** microcystin, contamination, toxicity, agriculture, health

## Abstract

Microcystins are natural hepatotoxic metabolites secreted by cyanobacteria in aquatic ecosystems. When present at elevated concentrations, microcystins can affect water quality aesthetics; contaminate drinking water reservoirs and recreational waters; disrupt normal ecosystem functioning; and cause health hazards to animals, plants, and humans. Animal and human exposures to microcystins generally result from ingesting contaminated drinking water or physically contacting tainted water. Much research has identified a multitude of liver problems from oral exposure to microcystins, varying from hepatocellular damage to primary liver cancer. Provisional guidelines for microcystins in drinking and recreational water have been established to prevent toxic exposures and protect public health. With increasing occurrences of eutrophication in freshwater systems, microcystin contamination in groundwater and surface waters is growing, posing threats to aquatic and terrestrial plants and agricultural soils used for crop production. These microcystins are often transferred to crops via irrigation with local sources of water, such as bloom-forming lakes and ponds. Microcystins can survive in high quantities in various parts of plants (roots, stems, and leaves) due to their high chemical stability and low molecular weight, increasing health risks for consumers of agricultural products. Studies have indicated potential health risks associated with contaminated fruits and vegetables sourced from irrigated water containing microcystins. This review considers the exposure risk to humans, plants, and the environment due to the presence of microcystins in local water reservoirs used for drinking and irrigation. Additional studies are needed to understand the specific health impacts associated with the consumption of microcystin-contaminated agricultural plants.

## 1. Introduction

Eutrophication is an environmental problem characterized by excessive algal and plant growth in aquatic ecosystems. Such a phenomenon largely occurs from agricultural nutrient runoff and continues to worsen due to global climate change, human population growth, and industrialization [1,2]. The symptoms of eutrophication have increased throughout coastal waters, drinking water reservoirs, estuaries, inland lakes, and slow-moving rivers, resulting in hypoxia, poor water quality, and noxious harmful algal blooms [2,3,4]. Harmful algal blooms, such as cyanobacterial blooms, commonly occur in eutrophic waters and may cause unsightly blue-green hues and release toxic secondary metabolites into the surrounding environment. Multiple environmental factors can promote cyanobacterial bloom formation and cyanotoxin production including environmental pollutants, light intensity, nutrients, salinity, pH, temperature, trace metals, and ultraviolet radiation [5].

Microcystins are the most prevalent and studied cyanotoxins within freshwater cyanobacterial blooms [6]. These biotoxins contain a rare 3-amino-9-methoxy-2,6,8-trimethyl-10-phenyl-4,6-decadienoic acid (ADDA) with two variable L amino acid residues [7]. The literature confirms over 300 variants of microcystin [8], with microcystin-leucine arginine (MC-LR) as the most important one due to its global distribution, ubiquity, and toxicity [9]. Multiple bloom-forming species (toxigenic and non-toxigenic) can produce MC-LR although *Microcystis* remains its primary manufacturer during cyanobacterial bloom incidents [10,11]. The presence of MC-LR in the environment constitutes an ecological and human health risk as concentration levels may exceed provisional limits in drinking (1 µg/L) and recreational waters (10 µg/L) [12].

The consumption of contaminated drinking water, aerosol inhalation, and dermal contact represent the main pathways of exposure to microcystins [13]. Additional exposure pathways, though less common, include the ingestion of contaminated algae dietary supplements, aquatic organisms, and fruits and vegetables [14]. Many studies have identified potential associations between microcystin exposure and liver disease. Hepatotoxic microcystins were found to accumulate in the tissue of dialysis patients in a Brazilian treatment center, many of whom died of subsequent liver failure [15]. Fishermen who frequented freshwater systems were also found to have well above the World Health Organization’s recommended amount of microcystins within their bloodstream (2.2–3.9 ng/mL) [16]. Freshwater microcystins in drinking water sources were identified as one potential risk factor for primary liver cancer in endemic China [17]. Similarly, in central Serbia, freshwater toxic cyanobacterial blooms releasing microcystins correlated with primary liver cancer incidence and mortality [18].

Microcystins are potent hepatotoxins and inhibit serine and threonine phosphatases in living organisms including animals and plants [19,20]. MC-LR is the most common and toxic variant of the microcystins; it traverses the cell membrane into hepatocytes through organic anion-transporting polypeptides (OATPs) [21]. MC-LR uptake is mediated by OATP1B1, OATP1B3, OATP1A2, and OATP1B2, and many of these OATPs are expressed in various organs (brain, kidney, small and large intestines, stomach), indicating the possible extensive toxicity of MC-LR [22]. MC-LR in mice has been demonstrated to cause alterations in mitochondrial membrane permeability and apoptosis [23], and prolonged exposure can suppress hematopoiesis function, leading to normocytic anemia [24].

Eutrophic waters containing hazardous microcystin concentrations has caused great concern for the agricultural industry. Repeated use of microcystin-contaminated water can alter the nutritional quality of crops, contaminate agricultural soils and plants, inhibit plant growth, and reduce crop production and yield [25]. For example, plant exposure to microcystins has been found to negatively impact tissue organization, cytoskeleton and membrane integrity, and the ability to uptake nutrients and manufacture sugars [25]. Rice seedlings exposed to microcystins of 100 µg/L were significantly affected in their growth and height, adding to the dry weights of leaves and roots [26]. A study examined the effects of microcystin buildup in agricultural crops, finding cabbage, dill, and parsley plants accumulated threefold greater amounts of microcystins compared to fruiting crops [27]. Another study observed a direct correlation between the amount of irrigation water used to water leafy vegetables and microcystin-contaminated crop soils, with leafy vegetables having 1.2–3.3 times more water used for irrigation purposes [28].

Lakes, streams, rivers, lagoons, and reservoirs account for the main sources of irrigation and drinking water worldwide. Microcystin variants have been detected in all these water reservoirs [29], increasing the potential likelihood of biotoxin transport into crop and drinking water systems. Microcystins have also been found in groundwater samples [30], which account for half of the drinking water available on Earth [31,32]. With excess fertilizer and nutrient use over the past few decades [33], the number of cyanobacteria and their associated toxins in eutrophic waters is expected to increase, suggesting more significant and persistent toxic harmful algal blooms. Agricultural water use from eutrophic sources has significantly impacted over 160 terrestrial food crop species [34]. Leafy greens, such as lettuce, spinach, watercress, and culinary herbs show the greatest uptake of microcystins into tissue most likely to be used in food preparation worldwide [35].

The purpose of the review is to reveal the persistence of toxic microcystins in water reservoirs and crops soils and their resultant impacts on agricultural plants and public health. The review is presented in chronological fashion to understand the interrelationships between water, soil, crops, and health in the context of microcystins. We first describe microcystin contamination in surface water, groundwater, and soil, followed by the most common input pathways that the cyanotoxins depend on for bioaccumulation in crops. Next, we cover the biosynthetic origin, mechanism of action, and toxicity of microcystins at the ecosystem and cellular levels. The review also considers the most recent works on toxic microcystins in agricultural plant parts and their potential health risks in humans, with the latter necessitating future studies.

## 2. Microcystins

The microcystins are perhaps the most widespread cyanotoxins in the natural environment and account for a maximum dry weight of 1% in terms of cyanobacterial mass [36]. Microcystin production is stimulated by important environmental factors such as light intensity, pH, temperature, trace metals, and ratio of total nitrogen to total phosphorus [5]. Microcystins belong to a family of monocyclic heptapeptides with ADDA and Mdha (*N*-methyldehydroalanine) binding moieties and two variable sites of amino acids. These structural components facilitate the binding of microcystin congeners to protein phosphatases in organisms while the variable sites influence toxicity [37]. MC-LR remains the priority congener in the environment and causes acute and chronic health effects in humans [12]. The environmental fate of microcystins remains unclear but influences the relationship with ecological and human health [38]. Contamination of water supplies and soil may provide insight into the environmental fate of microcystins relative to agricultural impacts and human health risks.

## 3. Contamination

### 3.1. Surface Water

The occurrence of microcystins in surface waters at hazardous levels can negatively affect ecosystem health. Microcystin concentrations generally remain below 100 µg/L in microcystin-polluted waters, although environmental conditions (nutrients, pH, and water temperature) can encourage concentrations greater than 1000 µg/L. The abundance of *Microcystis* morphospecies in waters has contributed to elevated microcystin concentrations [39,40,41]. *Microcystis aeruginosa* is reported as the high microcystin-producing morphospecies, followed by *Microcystis viridis* (low) and *Microcystis wesenbergii* (non-microcystin-producing) [42,43]. Microcystin contaminants are resistant to enzymatic degradation, high temperatures, pH alterations, and ultraviolet radiation owing to their heterocyclic structure [44,45]. Therefore, microcystin stability may facilitate transport into groundwater and agricultural soils in areas enduring cyanobacterial blooms.

### 3.2. Groundwater

After microcystins gradually accumulate in soil, they are released and migrate to lower soil horizons following precipitation events. Upon reaching deep soil layers, microcystins percolate into groundwater supplies and impair drinking water quality [46]. However, it has been reported that the use of contaminated groundwater wells for agricultural irrigation results in microcystin bioaccumulation among vegetable plants. A study in Saudi Arabia revealed microcystin levels in groundwater wells surpassing the World Health Organization’s guidance value of 1.0 µg/L, along with vegetable leaves and roots containing levels up to 1.2 µg/g fresh weight [30]. Microcystins from toxic cyanobacterial blooms accounted for a high concentration of 1.07 µg/L in a groundwater well hundreds of meters from the western coast of Lake Chaohu, China, an important source of water for drinking, washing, and irrigation [31]. These studies imply the hazardous nature of microcystins and their implications for agricultural practices, especially when they accumulate in crop soils.

### 3.3. Soil

Soil contamination from microcystins can disturb essential microorganisms of crop soil, which can accelerate toxin bioaccumulation in agricultural plants. Oxidative stresses caused by medial lethal concentrations of MC-LR (filter paper test = 0.149 µg/cm; acute soil test = 0.460 mg/kg) were discovered to exert profound impacts on the hatchability and survival of earthworms [47]. Frequent exposure of sediment bacteria to MC-LR was shown to inhibit the overall carbon metabolic activity of a microbial community [48]. These findings indicate the toxic effects of MC-LR on soil microorganisms, a critical biotic component involved in plant growth and development. Bioaccumulation in plants occurs after microcystin uptake, and the amount and time of absorption into edible plant tissue raises a food security concern [49,50]. Additionally, limited knowledge exists regarding the impact of microcystins on bacteria, fungi, and other essential organisms associated with plant growth. Although soil assists in microcystin uptake, the direct factors affecting its bioaccumulation in plants remain unknown [50]. Therefore, an understanding of the effects of microcystin input pathways through soils on crop bioaccumulation and toxicity, considering variations in the bioavailability and speciation of microcystins, may be beneficial.

## 4. Input Pathways

### 4.1. Irrigation with Polluted Water

Irrigation with polluted water is the primary route of introduction of cyanotoxins (microcystins) in agricultural soils. Extracellular microcystins within irrigation water may leach into soils from constant spray, increasing the bioavailability of toxin [51]. Two studies indicated that contaminated irrigation water with microcystins threaten crop quality and yield [52,53]. One study confirmed high uptake values of MC-LR and microcystin-leucine phenylalanine (MC-LF) in the roots of alfalfa and wheat (1.3 mg/kg dry weight) [52]. A greenhouse experiment measured microcystin content in the roots of clover, lettuce, rape, and ryegrass, with clover presenting the highest total microcystin concentrations (1.45 mg/kg dry weight). Since microcystins can bioconcentrate in plant tissue, they can enter the animal and human food chains at concentrations above the recommended tolerable limits [53]. The tolerable daily intake value for chronic exposure to MC-LR is 0.04 µg/kg body weight, as proposed by the World Health Organization [12].

### 4.2. Application of Cyanobacterial Manure

Cyanobacterial fertilizer, which contains living cyanobacteria cultures that aid in harnessing solar energy, nutrients, and water resources essential to plant growth have become popular in recent years [54,55]. The application of cyanobacterial fertilizer in tandem with manure has the potential to improve soil quality and plant growth and reduce crop production costs in agriculture [55]. However, its use in soils affected by cyanobacterial blooms is not recommended due to higher bioaccumulation capacity in edible plants from polluted soil [50]. Intracellular microcystins are contained in cyanobacterial manure, and cell lysis can facilitate the bioavailability of microcystins in soil. Furthermore, the co-existence of microcystin congeners in manure is considered a human health risk [56]. These cyanotoxins have been associated with tumor promotion in animals and liver and colon cancer in humans [57]. Careful and proper application of cyanobacterial manure in soils may improve agricultural plant quality and protect public health from contaminated agricultural foods.

### 4.3. Compost

Compost, a mixture containing decaying organic matter has traditionally been used as a soil conditioner and fertilizer to aid vigorous plant growth. The agricultural use of compost has risen in the past decade to foster improved soil ecosystem health for greater crop outputs with less dependence on chemical inputs [58]. While the negative influence of microcystin contamination is well documented for marine invertebrates, few studies have indicated its effect on soil-dwelling species [47,59]. Soil nematodes were shown to have a reduction in overall lifespan and a decrease in reproduction and were less motile in the presence of a mere 1.0 µg/kg MC-LR concentration, with any higher dose largely obliterating nematode existence [59]. While this might be a positive for the control of harmful plant-feeding species, the broad-spectrum nature means it is equally problematic for beneficial species [47]. Earthworms who digest large quantities of organic matter in the soil layer are more likely to absorb high concentrations of MC-LR [47]. Interestingly, when compost levels decreased in total concentration, earthworms continued to show high levels, perhaps due to bioaccumulation.

Cyanobacteria have demonstrated promise in the removal of harmful pathogens in manure and compost; however, microcystin toxins reduce the overall activity of beneficial bacteria and fungi involved in nitrogen fixation [60]. Compost used for crop production has increased interest in the likelihood of unintentional contamination [61]. As has been the case for pesticides, leafy greens were most vulnerable to the accumulation of hazardous levels of microcystin, representing a potential health risk to consumers [62]. Higher mean concentrations of MCs, in the 12.3–22.8 µg/kg range, were found in lettuce and cabbage, as compared to root vegetables such as carrot (10.5–12.6 µg/kg) that were grown in compost-rich soil [28]. The results imply possible health concerns in agricultural areas where compost material is preferred over other soil applicators and fertilizers.

## 5. Toxicity

### 5.1. Biosynthesis

Microcystins are toxic peptides of non-protein amino acids encoded by non-ribosomal peptide synthetase (NRPS)/polyketide synthase enzymes [63]. Genetic analysis has indicated that a large NRPS gene cluster is involved in promoting microcystin biosynthesis. DNA sequence results from *Microcystis* strain K-139 identified an *mycABC* operon and revealed a microcystin synthetase gene operon (*mycD*, *mycE*, *mycF*, *mycG*) within the NRPS gene cluster [63]. NRPS enzymes use a thiotemplate mechanism to catalyze the synthesis of peptides in prokaryotes and lower eukaryotes. Many cyclic peptides are synthesized by NRPS enzymes including the antimicrobial agents, cyclosporine, penicillin, and vancomycin. NRPS products are highly diverse in chemical structure, owing to incorporated hydroxylated and methylated amino acids [64]. Covalent modifications of amino acid residues may explain the myriad of microcystin variants detected in freshwater sites, some of which present a danger to aquatic and terrestrial plants.

### 5.2. Mechanism of Action

Microcystins are cyclic heptapeptides with a chemical structure of Adda-D-Glu-Mdha-D-Ala-L-X-D-MeAsp-L-Z, where X and Z refer to variable sites of amino acid residues [64]. Microcystin-leucine arginine (MC-LR) is the most prevalent and toxic of all microcystin congeners [9]. The mechanism of action of MC-LR involves the inhibition of serine and threonine phosphatases [19,20]. Microcystins exert a strong affinity toward protein phosphatase 1 (PP1) and 2A (PP2A) and, to a lesser extent, protein phosphatase 2B (PP2B) [65]. A two-step mechanism occurs between microcystin and PP1 and PP2A, wherein microcystin inactivates catalytic subunits (PP1-c and PP-2ac) through rapid binding, followed by an extended period of covalent interaction [66]. Accessibility to PP1-c’s (catalytic subunit) active site is blocked when the carboxyl terminus of MeAsp interacts with Arg96 and Tyr134 [67]. When its active site is blocked, PP1 can no longer bind to its substrates, preventing the removal of phosphates from these molecules, which are critical to maintaining cellular homeostasis in biological organisms. MC-LR toxicity has been well studied, as supported by changes in liver structure and cytoskeleton deformities in animals and plants, respectively. The following section describes some recent findings on the adverse effects of toxic microcystins in the local environment as well as in plants.

### 5.3. Ecotoxicity

Microcystins are secondary metabolites produced by cyanobacteria in the aquatic environment. The exposure of aquatic organisms to microcystins has been shown to alter behavior, cause bioaccumulation in tissues, inhibit growth, and reduce fecundity and survivorship [68]. Toxic microcystins have resulted in episodes of fatality to aquatic biota, livestock, and wild animals worldwide [69]. The biological detoxification pathways of resident organisms are major determinants of microcystin toxicity in the ecosystem [38]. Biotransformation of microcystins into conjugate intermediates via the glutathione pathway is conserved in many aquatic organisms, which may offer protection from toxicologic effects of microcystins. The metabolic process serves to eliminate microcystins from the organism and release them back in the natural environment [38]. However, toxin release from exposed organisms can harm other aquatic organisms and humans as microcystins transfer to higher trophic levels in the food chain. Microcystins can also leach into groundwater and transfer into soils for agricultural production [46]. Thus, we present the human health risks associated with microcystins in waters sourced for irrigation and crop soils in agriculture.

### 5.4. Phytotoxicity

Phytotoxicity, a delay in overall seed germination, inhibition of plant growth, or other adverse effects on plant growth and development, has been attributed to the presence of microcystins [62]. Dephosphorylation is a critical process of cell cycle control and protein modification in living organisms, including higher plants. However, the inhibition of dephosphorylation can negatively affect cell function, causing hyperphosphorylation among cytokeratin proteins and significant alterations in cytoskeletal elements [70,71]. More precisely, microcystins can associate with F-actin and microtubules, altering F-actin organization and disorienting microtubules in plant cells [72]. This directly impacts photosynthesis activity, with overall chlorophyll concentration decreasing as much as 0.80 mg/g [73]. Stomatal integrity is similarly impacted by the weakening cytoskeletal elements and can lead to oxidative stress including less leaf transpiration and poor gas exchange [74]. Roots also indicated adverse influence, with low concentrations (<250 µg/L) causing an overall reduction in the ability to uptake necessary nutrients. Microcystin concentrations above 250 µg /L caused total inhibition of assemblage of nutrients at the root level [75]. The metabolism of nitrogen, an essential element for plant growth, shows significant decrease when aquatic and terrestrial plants are placed in concentrations of 9.9–29.8 µg/L of microcystin for 30 days. Enzymatic activity that assists in sequestering nitrogen into tissue, including glutamic-pyruvic transaminase and glutamic-oxaloacetic transaminase, are reduced at this concentration level [76].

## 6. Agricultural Plants

Plant growth, whether in terrestrial or aquatic form, has shown sensitivities to microcystins [62,77,78,79]. The two are distinguished from each other by the traditional definition of true aquatic plant, meaning it must be submersed in water for most of its life cycle [74]. Terrestrial plants, in contrast, spend their life cycle on land and root directly into the soil [80]. Those that can withstand periods of standing water are still classified as terrestrial, land-dwelling plants [80]. Of the two, terrestrial plants have received significant attention where crops are concerned, as those are directly consumed [25,62]. The effects on tissue and seedling growth are presented below (Table 1).

### 6.1. Plant Seedling Growth

Rigorous regulation of microcystin-contaminated water use for irrigation is not actively practiced worldwide. As a result, entire crop networks are susceptible to potentially toxic bacteria, including cyanobacteria, which may directly impact the human food system. In the past decade, studies have measured the effects of varying concentrations of microcystins on the growth of agriculturally important crops, including leafy greens, herbs, root vegetables, and squash [34,53,62,90,91]. Height, biomass, leaf surface area, seedling diameter, and root development were significantly reduced across all crops (root vegetables, herbs, leafy greens, and squash) tested, with leafy greens holding the highest risk to exposure [77]. While all vegetable and fruit crops studied demonstrated negative impacts, the seedling stage appeared the most vulnerable to uptake of microcystins into developing tissue. *Rhizobia* nodules, which are essential for many growing seedlings to successfully fix and uptake nitrogen, are reduced or non-existent when grown in high concentrations of microcystins [92]. As a result, root surface area, length, number of roots present, and total biomass are reduced by upward of 60% in leafy green vegetables [79].

### 6.2. Tissue Growth

Irrigating mature crops with microcystin-contaminated water also leads to poor nitrogen fixation, reduced height, and reduction in total biomass in tissue development [77,78,79]. As seen with seedlings, the higher the concentration of microcystins present in irrigation water, the more at-risk tissues are for reduced photosynthesis, decreased leaf and root production, and an overall reduction in biomass [74,77,78,79,80]. In a survey of 35 crop species ranging from leafy greens to root vegetables, all reported losses of at least 30% in total leaf surface area [79]. Further analysis suggests that microcystins play a direct role in the inhibition of photosynthetic activities and can cause tissue death, increase oxidative stress, reduce membrane integrity, and impair the ability of roots to absorb nutrients [85,88,93]. Anomalies in tissue structure were apparent in crops grown hydroponically, with high concentration of microcystins (10 µg/L and 20 µg/L) [94]. Additional alterations, such as slowing of mitotic and metabolic processes and reduced development, increased with the increase of concentration [82,86].

Despite the effects of microcystins on seedling and tissue growth, they are not the sole contributing factor in plant developmental changes [25]. The impacts of microcystins are greatest at higher concentrations, with the greatest effect on the seedling stage [77,78,81,84]. Due to the structure, surface area, and gas exchange exhibited by leafy greens, microcystins can readily diffuse through stomata into plant tissue and cause negative impacts [79,93,95,96]. Hydroponically grown species of leafy greens including watercress, lettuce, and spinach, over those grown in soil-based cultures, were 30% more likely to have hinderance of growth [97,98]. A study identified root growth inhibition within *Lactuca sativa* (lettuce) from exposure to *Microcystis aeruginosa* strains [99]. Photosynthesis activity within *Lycopersicon esculentum* leaves was not affected when exposed to a pure concentration of MC-LR (100 µg/L) [100]. Additional analysis is needed to determine whether microcystin concentrations in irrigation water used for hydroponically grown lettuce and herbs pose a risk to human health.

### 6.3. Aquatic Plants

Microcystins can readily enter many aquatic plant species through simple diffusion, stomata of leaves in contact with water, and absorption via the root system [87]. Several cases have documented accumulated microcystin uptake in both field and laboratory settings [101,102]. Submerged species show the highest concentrations present in tissue, with *Elodea canadensis* showing a concentration of 40 µg/g of dry weight [103]. *Lemna minor* (duckweed) and *Wolffia arrhizal* (duckweed) demonstrated a reduction in growth over the course of a 5-day exposure study [102]. Clearly, method of uptake, plant species, concentration, and type of microcystins present, and length of exposure play a significant role in determining accumulation (Table 2).

While microcystin accumulation proves harmful to essential processes such as photosynthesis, overall height, and weight of the mature plant, aquatic species can detoxify the bacteria through the action of a natural antioxidant, glutathione S-transferases (GSTs) (Table 1). High levels of GSTs found in contaminated plant tissue suggests that microcystin presence may trigger their production [104]. Laboratory studies of several different species show similar results, signaling the likelihood of microcystins contributing to the enhancement of GST concentrations in exposed aquatic plants [38,105]. In a timed-exposure study of cyanobacteria and microcystins, Lemnaceae sp. (duckweed), experienced increased concentrations of GSTs present within a 24 h timespan [90]. In another timed-exposure study, similar effects were seen in *Vallisneria natans* [91]. Perhaps most impressive, bioaccumulation of microcystin in *Lemna minor* after 5 days of exposure showed resistance to a concentration level (3.0 µg/L) that had earlier been detrimental [106]. More studies are needed to explore the possible implications of GST for resistance against microcystins.

**Table 2:** Accumulation of microcystins in aquatic plants.

Species	Environment	Mode of Uptake	* Microcystin Toxins	** Concentration of Microcystin	*** Plant Response	Reference
*Alternanthera philoxeroides* (alligator weed)	Submerged	Root absorption Diffusion	Total MCs	169–3945 ng/g	--	[107]
*Ceratophyllum dermersum* (hornwort)	Submerged	Root absorption Diffusion	MC-LR	71 µg/g	--	[103]
*Elodea canadensis* (American waterweed)	Submerged	Root absorption Leaf contact with water surface Diffusion	MC-LR	40 µg/g	--	[103]
*Hydrilla verticillata* (water thyme)	Submerged	Root absorption Diffusion	Total MCs MC-LR MC-RR MC-YR	>1000 µg/kg >1000 µg/kg <500 µg/kg <500 µg/kg	Biotransformation of MCs	[108]
*Lemna gibba* (duckweed)	Floating	Root absorption Leaf contact with water surface	MC-LR	2.44 µg/g	Reduction in plant growth and chlorophyll content Biotransformation of MCs	[109]
*Phragmites australis* (common reed)	Floating	Root absorption Leaf contact with water surface	MC-LR	5–40 µg/L	Inhibition of growth and development	[110]
*Polygonum portorcensis* (smooth smartweed)	Submerged	Root absorption Diffusion	Total MCs MC-LR MC-RR MC-YR	>400 µg/kg >400 µg/kg <200 µg/kg <200 µg/kg	Biotransformation of MCs	[108]
*Trapa natans* (water chestnut)	Floating	Root absorption Leaf contact with water surface	Total MCs	1.68 ng/g	--	[111]
*Typha* sp. (cattail)	Floating	Root absorption Leaf contact with water surface	Total MCs MC-LR MC-RR MC-YR	>1500 µg/kg >1500 µg/kg <500 µg/kg <500 µg/kg	Biotransformation of MCs	[108]
*Vallisneria natans* (eelgrass)	Submerged	Root absorption Diffusion	MC-RR	10 mg/L	Reduction in root and leaf numbers	[105]

* Total MCs = concentration of analyzed MC congeners; MC-LR = microcystin-leucine arginine; MC-RR = microcystin-arginine arginine; MC-YR = microcystin-tyrosine arginine. ** Concentration of microcystin is expressed as ng/g, µg/g, µg/kg, or mg/L. Reported concentrations should be interpreted with caution. *** -- = no information available.

## 7. Human Health Risks

Epidemiological research has indicated potential health effects of microcystins from human consumption of contaminated drinking water and aquatic foods [15,16,17,18]. A recent work posits environmental microcystins as an emergent risk factor in endemic regions plagued by hepatocellular carcinoma [112]. It is possible that these regions continually rely on bloom-forming water reservoirs for drinking water, food, and irrigation. Nevertheless, little remains known about the human health risks of microcystins in agricultural products. Given the discussed high levels of microcystin accumulation in leafy vegetables, including lettuces and spinach when grown through use of contaminated irrigation, the possibility of health risk is present [50,53,74,77,78,79,91,93,98,99,104]. Since leafy greens are now popular to grow hydroponically, their sensitivity to microcystins and other bacteria uptake needs to be studied and taken into serious consideration from a health standpoint. This also raises questions and concerns regarding the potential health risks to agricultural workers who are directly in contact with irrigation water and thus exposed to high microcystin concentrations [77,78].

## 8. Implications for Future Research

The overall findings of this review depict a clear presence of the microcystin in drinking water and crop systems, raising concerns of health risk and food safety. This review indicates a need for further research in areas experiencing anthropogenic eutrophication of water reservoirs. The increase in microcystins in enriched water sources used for irrigation and consumption raises concern over the health risks posed to nearby communities. Studies examining soil- and hydroponic-based agricultural systems have indicated plant sequestering of microcystins into plant tissue, with leafy greens showing the highest numbers. More research is needed, particularly on a larger scale, to further examine the relationship of microcystins with hydroponic, in soil, and laboratory-based conditions. At present, few studies have explored the presence of microcystins in media amendments such as manure and compost, which could also add to the number of toxic microcystins present in agricultural plants. Although drinking water contamination from microcystin appears well studied, the food safety risks associated with the irrigation of agricultural crops, both in small- and large-scale agriculture, could benefit from additional review. Several studies indicate the possibility of microcystins eliciting a response to release glutathione S-transferases to detoxify potential negative effects of the toxin. More research in this arena could provide more insight into how this could be employed in large-scale detoxification of water used in environmental and agricultural contexts.

## Figures and Tables

**Table 1:** Physiological effects in agricultural plants from various microcystin exposure routes.

Species	Experimental Design	Concentration of Microcystin *	Duration of Exposure (Days)	Stage of Development	Physiological Effects	Reference
*Brassica juncea* (mustard green)	Pot study	150 µg/kg MC-LR	10 d	Mature plants	Reduced plant height and weight	[56]
*Brassica napus* (rape seed)	Germination	600–3000 µg/L MC-LR	10 d	Seeds	Reduced germination	[81]
*Daucus carota* (carrot)	Independent exposure experiment	50 µg/L MC-LR	28 d	Mature plants	Reduced root growth Increased photosynthetic efficiency	[82]
*Ipomoea batatas* (sweet potato)	Pot study	150 µg/kg MC-LR	10 d	Mature plants	Reduced plant height and weight	[56]
*Lactuca sativa* (lettuce)	Hydroponics Hydroponics	50 µg/L MCs 100 µg/L MC-LR	21 d 10 d	Mature plants Mature plants	Reduced leaf growth and mineral content Reduced biomass of leaves and mineral content Increased GST activity in roots	[77,78]
*Lepidium sativum* (watercress)	Germination	10 µg/L MC-LR	6 d	Seeds	Reduced radicle length and shoot weight	[83]
*Medicago sativa* (alfalfa)	Germination	5 µg/L MCs	7 d	Seedlings	Inhibition of germination and root growth Increased lipid peroxidation	[84]
*Oryza sativa* (rice)	Hydroponics Hydroponics	1–3000 µg/L MC 500 µg/L MCs	7 d 30 d	Seedlings	Reduced biomass of leaves, stems, and roots Reduced root weight, length, surface area and volume Increased levels of tartaric acid and malic acid	[26] [85]
*Phaseolus vulgaris* (green bean)	Germination	3500 µg/L MC-LR	30 d	Seeds	Reduced chlorophyll content, delayed development Reduced conductivity and phototropic response	[86]
*Solanium lycopersicum* (tomato)	Soil	5 µg/L MC-LR	90 d	Seeds	Stimulation of inflorescence and blooming of flower	[87]
*Spinacia oleracea* (spinach)	Hydroponics	50 µg/L MCs	21 d	Mature plants	Reduced leaf growth and mineral content	[77]
*Triticum aestivum* (wheat)	Germination Soil	0.5 µg/L MC-LR	3 d 14 d	Seeds	Reduced germination Reduced photosynthesis and root and shoot development Increased GST activity	[88]
*Zea mays* (corn)	Germination	100,000–800,000 µg/L	1 d	Seeds	Reduced plant height and weight	[89]

* Concentration of microcystin exposure on crop species is expressed as µg/L or µg/kg. MCs = total concentration of microcystin (variants not available) MC-LR = microcystin-leucine arginine; GST = glutathione-S-transferase.

## Data Availability

No new data were created or analyzed in this study. Data sharing is not applicable to this article.
