# Correction: Melaram et al. Microcystin Contamination and Toxicity: Implications for Agriculture and Public Health. *Toxins* 2022, *14*, 350

**DOI:** 10.3390/toxins17070320

**Published:** 2025-06-24

**Authors:** Rajesh Melaram, Amanda R. Newton, Jennifer Chafin

**Affiliations:** Department of Science, Eastern Florida State College, Melbourne, FL 32935, USAchafin.jennifer@titans.easternflorida.edu (J.C.)

The authors wish to make the following corrections to their paper [1].

## Error in Content

### 5.1. Biosynthesis

The terms “ecosystem” “agricultural” were mistakenly introduced during the last sentence. The corrected statement is: 

“Covalent modifications of amino acid residues may explain the myriad of microcystin variants detected in freshwater sites, some of which present a danger to aquatic and terrestrial plants.”

### 5.4. Phytotoxicity

The letter “s” was missing after the word “effect” in the first sentence. The corrected statement is:

“Phytotoxicity, a delay in overall seed germination, inhibition of plant growth, or other adverse effects on plant growth and development, has been attributed to the presence of microcystins.”

The concentration of microcystin and names of enzymes were incorrect, and the duration of exposure was missing in the last two sentences. The enzymatic activity is modified. The corrected statements are: 

“The metabolism of nitrogen, an essential element for plant growth, shows significant decrease when aquatic and terrestrial plants are placed in concentrations of 9.9–29.8 µg/L of microcystin for 30 days. Enzymatic activity that assists in sequestering nitrogen into tissue, including glutamic-pyruvic transaminase and glutamic-oxaloacetic transaminase, are reduced at this concentration level [76].”

### 6.2. Tissue Growth

The number of crop species was numerically incorrect in the third sentence. The corrected statement is: 

“In a survey of 35 crop species ranging from leafy greens to root vegetables, all reported losses of at least 30% in total leaf surface area [79].”

The second treatment of microcystin concentration was missing. The corrected statement is:

“Anomalies in tissue structure were apparent in crops grown hydroponically, with high concentration of microcystins (10 µg/L and 20 µg/L) [94].”

The word “alternations” was used instead of “alterations.” The corrected statement is:

“Additional alterations, such as slowing of mitotic and metabolic processes and reduced development, increased with the increase of concentration [82,86].”

### 6.3. Aquatic Plants

Incorrect reporting of concentrations occurred in the third sentence of the first paragraph and the second-to-last sentence of the second paragraph. The two corrected statements are:

“Submerged species show the highest concentrations present in tissue, with *Elodea canadensis* showing a concentration of 40 µg/g of dry weight [103].”

“Perhaps most impressive, bioaccumulation of microcystin in *Lemna minor* after 5 days of exposure showed resistance to a concentration level (3.0 µg/L) that had earlier been detrimental [106].”

## Error in Tables

Several errors were noted in Tables 1 and 2. Major errors in Table 1 included incorrect microcystin congeners and reported concentrations. Minor errors in Table 1 included the misspelling of the genus “*Daucus*”, misclassification of “Stage of Development” and “Route of Exposure” for crop species, and mismatched “Physiological Effects”. Minor errors in Table 2 included misclassification of “Environment” in addition to discrepancies in “Mode of Uptake” and “Plant Response”. The corrections, which were not fully addressed during peer review, are presented below. 

**Table 1:** Physiological effects in agricultural plants from various microcystin exposure routes.

Species	Experimental Design	Concentration of Microcystin *	Duration of Exposure (Days)	Stage of Development	Physiological Effects	Reference
*Brassica juncea* (mustard green)	Pot study	150 µg/kg MC-LR	10 d	Mature plants	Reduced plant height and weight	[56]
*Brassica napus* (rape seed)	Germination	600–3000 µg/L MC-LR	10 d	Seeds	Reduced germination	[81]
*Daucus carota* (carrot)	Independent exposure experiment	50 µg/L MC-LR	28 d	Mature plants	Reduced root growth Increased photosynthetic efficiency	[82]
*Ipomoea batatas* (sweet potato)	Pot study	150 µg/kg MC-LR	10 d	Mature plants	Reduced plant height and weight	[56]
*Lactuca sativa* (lettuce)	Hydroponics Hydroponics	50 µg/L MCs 100 µg/L MC-LR	21 d 10 d	Mature plants Mature plants	Reduced leaf growth and mineral content Reduced biomass of leaves and mineral content Increased GST activity in roots	[77,78]
*Lepidium sativum* (watercress)	Germination	10 µg/L MC-LR	6 d	Seeds	Reduced radicle length and shoot weight	[83]
*Medicago sativa* (alfalfa)	Germination	5 µg/L MCs	7 d	Seedlings	Inhibition of germination and root growth Increased lipid peroxidation	[84]
*Oryza sativa* (rice)	Hydroponics Hydroponics	1–3000 µg/L MC 500 µg/L MCs	7 d 30 d	Seedlings	Reduced biomass of leaves, stems, and roots Reduced root weight, length, surface area and volume Increased levels of tartaric acid and malic acid	[26] [85]
*Phaseolus vulgaris* (green bean)	Germination	3500 µg/L MC-LR	30 d	Seeds	Reduced chlorophyll content, delayed development Reduced conductivity and phototropic response	[86]
*Solanium lycopersicum* (tomato)	Soil	5 µg/L MC-LR	90 d	Seeds	Stimulation of inflorescence and blooming of flower	[87]
*Spinacia oleracea* (spinach)	Hydroponics	50 µg/L MCs	21 d	Mature plants	Reduced leaf growth and mineral content	[77]
*Triticum aestivum* (wheat)	Germination Soil	0.5 µg/L MC-LR	3 d 14 d	Seeds	Reduced germination Reduced photosynthesis and root and shoot development Increased GST activity	[88]
*Zea mays* (corn)	Germination	100,000–800,000 µg/L	1 d	Seeds	Reduced plant height and weight	[89]

* Concentration of microcystin exposure on crop species is expressed as µg/L or µg/kg. MCs = total concentration of microcystin (variants not available) MC-LR = microcystin-leucine arginine; GST = glutathione-S-transferase.

**Table 2:** Accumulation of microcystins in aquatic plants.

Species	Environment	Mode of Uptake	* Microcystin Toxins	** Concentration of Microcystin	*** Plant Response	Reference
*Alternanthera philoxeroides* (alligator weed)	Submerged	Root absorption Diffusion	Total MCs	169–3945 ng/g	--	[107]
*Ceratophyllum dermersum* (hornwort)	Submerged	Root absorption Diffusion	MC-LR	71 µg/g	--	[103]
*Elodea canadensis* (American waterweed)	Submerged	Root absorption Leaf contact with water surface Diffusion	MC-LR	40 µg/g	--	[103]
*Hydrilla verticillata* (water thyme)	Submerged	Root absorption Diffusion	Total MCs MC-LR MC-RR MC-YR	>1000 µg/kg >1000 µg/kg <500 µg/kg <500 µg/kg	Biotransformation of MCs	[108]
*Lemna gibba* (duckweed)	Floating	Root absorption Leaf contact with water surface	MC-LR	2.44 µg/g	Reduction in plant growth and chlorophyll content Biotransformation of MCs	[109]
*Phragmites australis* (common reed)	Floating	Root absorption Leaf contact with water surface	MC-LR	5–40 µg/L	Inhibition of growth and development	[110]
*Polygonum portorcensis* (smooth smartweed)	Submerged	Root absorption Diffusion	Total MCs MC-LR MC-RR MC-YR	>400 µg/kg >400 µg/kg <200 µg/kg <200 µg/kg	Biotransformation of MCs	[108]
*Trapa natans* (water chestnut)	Floating	Root absorption Leaf contact with water surface	Total MCs	1.68 ng/g	--	[111]
*Typha* sp. (cattail)	Floating	Root absorption Leaf contact with water surface	Total MCs MC-LR MC-RR MC-YR	>1500 µg/kg >1500 µg/kg <500 µg/kg <500 µg/kg	Biotransformation of MCs	[108]
*Vallisneria natans* (eelgrass)	Submerged	Root absorption Diffusion	MC-RR	10 mg/L	Reduction in root and leaf numbers	[105]

* Total MCs = concentration of analyzed MC congeners; MC-LR = microcystin-leucine arginine; MC-RR = microcystin-arginine arginine; MC-YR = microcystin-tyrosine arginine. ** Concentration of microcystin is expressed as ng/g, µg/g, µg/kg, or mg/L. Reported concentrations should be interpreted with caution. *** -- = no information available.

## Error in References

Numerous inconsistencies were evident in the original publication, such as missing, incorrect, or out-of-order citations. All citations have now been corrected. With this correction, the order of some references has been adjusted accordingly.

### References Citation Changes in Maintext (Reordered Labeled)

#### Section 1: Introduction

“A study examined the effects of microcystin buildup in agricultural crops, finding cabbage, dill, and parsley plants accumulated threefold greater amounts of microcystins compared to fruiting crops [27].”

“Microcystin variants have been detected in all these water reservoirs [29], increasing the potential likelihood of biotoxin transport into crop and drinking water systems. Microcystins have also been found in groundwater samples [30], which account for half of the drinking water available on Earth [31,32].”

“Agricultural water use from eutrophic sources has significantly impacted 160 terrestrial food crop species [34].”

#### Section 4.1: Irrigation with Polluted Water

“Two studies indicated that contaminated irrigation water with microcystins threatens crop quality and yield [52,53].”

#### Section 4.2: Application of Cyanobacterial Manure

“Cyanobacterial fertilizer, which contains living cyanobacteria cultures that aid in harnessing solar energy, nutrients, and water resources essential to plant growth have become popular in recent years [54,55]. The application of cyanobacterial fertilizer in tandem with manure has the potential to improve soil quality and plant growth and reduce crop production costs in agriculture [55]. However, its use in soils affected by cyanobacterial blooms is not recommended due to higher bioaccumulation capacity in edible plants from polluted soil [50].”

“Furthermore, the co-existence of microcystin congeners in manure is considered a human health risk [56].”

#### Section 4.3: Compost

“The agricultural use of compost has risen in the past decade to foster improved soil ecosystem health for greater crop outputs with less dependence on chemical inputs [58]. While the negative influence of microcystin contamination is well documented for marine invertebrates, few studies have indicated its effect on soil-dwelling species [47,59]. Soil nematodes were shown to have a reduction in overall lifespan and a decrease in reproduction and were less motile in the presence of a mere 1.0 µg/kg MC-LR concentration, with any higher dose largely obliterating nematode existence [59].”

“Compost used for crop production has increased interest in the likelihood of unintentional contamination [61].”

“Higher mean concentrations of MCs, in the 12.3–22.8 µg /kg were found in lettuce and cabbage, as compared to root vegetables such as carrot (10.5–12.6 µg/kg) that were grown in compost-rich soil [28].”

#### Section 5.2: Mechanism of Action

“Microcystins exert a strong affinity toward protein phosphatase 1 (PP1) and 2A (PP2A) and, to a lesser extent, protein phosphatase 2B (PP2B) [65].”

#### Section 5.4: Phyotoxicity

“This directly impacts photosynthesis activity, with overall chlorophyll concentration decreasing as much as 0.80 mg/g [73]. Stomatal integrity is similarly impacted by the weakening cytoskeletal elements and can lead to oxidative stress including less leaf transpiration and poor gas exchange [74].”

“Microcystin concentrations above 250 µg/L caused total inhibition of assemblage of nutrients at the root level [75].”

#### Section 6: Agricultural Plants

“Plant growth, whether in terrestrial or aquatic form, has shown sensitivities to microcystins [62,77–79]. The two are distinguished from each other by the traditional definition of true aquatic plant, meaning it must be submersed in water for most of its life cycle [74]. Terrestrial plants, in contrast, spend their life cycle on land and root directly into the soil [80]. Those that can withstand periods of standing water are still classified as terrestrial, land-dwelling plants [80]. Of the two, terrestrial plants have received significant attention where crops are concerned, as those are directly consumed [25,26]. The effects on tissue and seedling growth are presented below (Table 1).”

#### Section 6.1: Plant Seedling Growth

“In the past decade, studies have measured the effects of varying concentrations of microcystins on the growth of agriculturally important crops, including leafy greens, herbs, root vegetables, and squash [34,53,62,90,91]. Height, biomass, leaf surface area, seedling diameter, and root development were significantly reduced across all crops (root vegetables, herbs, leafy greens, and squash) tested, with leafy greens holding the highest risk to exposure [77].”

“*Rhizobia* nodules, which are essential for many growing seedlings to successfully fix and uptake nitrogen, are reduced or non-existent when grown in high concentrations of microcystins [92]. As a result, root surface area, length, number of roots present, and total biomass are reduced by upward of 60% in leafy green vegetables [79].”

#### Section 6.2: Tissue Growth

“Irrigating mature crops with microcystin-contaminated water also leads to poor nitrogen fixation, reduced height, and reduction in total biomass in tissue development [77–79]. As seen with seedlings, the higher the concentration of microcystins present in irrigation water, the more at-risk tissues are for reduced photosynthesis, decreased leaf and root production, and an overall reduction in biomass [74,77–80]. In a survey of 35 crop species ranging from leafy greens to root vegetables, all reported losses of at least 30% in total leaf surface area [79]. Further analysis suggests that microcystins play a direct role in the inhibition of photosynthetic activities and can cause tissue death, increase oxidative stress, reduce membrane integrity, and impair the ability of roots to absorb nutrients [85,88,93]. Anomalies in tissue structure were apparent in crops grown hydroponically, with high concentration of microcystins (10 µg/L and 20 µg/L) [94].”

“Despite the effects of microcystins on seedling and tissue growth, they are not the sole contributing factor in plant developmental changes [25]. The impacts of microcystins are greatest at higher concentrations, with the greatest effect on the seedling stage [77,78,81,84]. Due to the structure, surface area, and gas exchange exhibited by leafy greens, microcystins can readily diffuse through stomata into plant tissue and cause negative impacts [79,93,95,96]. Hydroponically grown species of leafy greens including watercress, lettuce, and spinach, over those grown in soil-based cultures, were 30% more likely to have hinderance of growth [97,98].”

#### Section 6.3: Aquatic Plants

“Microcystins can readily enter many aquatic plant species through simple diffusion, stomata of leaves in contact with water, and absorption via the root system [87]. Several cases have documented accumulated microcystin uptake in both field and laboratory settings [101,102]. Submerged species show the highest concentrations present in tissue, with *Elodea canadensis* showing a concentration of 40 µg/g of dry weight [103].”

“While microcystin accumulation proves harmful to essential processes such as photosynthesis, overall height, and weight of the mature plant, aquatic species can detoxify the bacteria through the action of a natural antioxidant, glutathione S-transferases (GSTs) (Table 1). High levels of GSTs found in contaminated plant tissue suggests that microcystin presence may trigger their production [104]. Laboratory studies of several different species show similar results, signaling the likelihood of microcystins contributing to the enhancement of GST concentrations in exposed aquatic plants [38,105].”

#### Section 7: Human Health Risks

“Epidemiological research has indicated potential health effects of microcystins from human consumption of contaminated drinking water and aquatic foods [15–18]. A recent work posits environmental microcystins as an emergent risk factor in endemic regions plagued by hepatocellular carcinoma [112].”

“Given the discussed high levels of microcystin accumulation in leafy vegetables, including lettuces and spinach when grown through use of contaminated irrigation, the possibility of health risk is present [50,53,74,77–79,91,93,98,99,104].”

“This also raises questions and concerns regarding the potential health risks to agricultural workers who are directly in contact with irrigation water and thus exposed to high microcystin concentrations [77,78].”

### Adjusted References (Reordered Labeled)

#### Switch Original Refs 16 and 17

- 16. Chen, J.; Xie, P.; Li, L.; Xu, J. First identification of the hepatotoxic microcystins in the serum of a chronically exposed human population together with indication of hepatocellular damage. *Toxicol. Sci.* **2009**, *108*, 81–89.
- 17. Ueno, Y.; Nagata, S.; Tsutsumi, T.; Hasegawa, A.; Watanabe, M.F.; Park, H.D.; Chen, G.C.; Yu, S.Z. Detection of microcystins, a blue-green algal hepatotoxin, in drinking water sampled in Haimen and Fusui, endemic areas of primary liver cancer in China, by highly sensitive immunoassay. *Carcinogenesis* **1996**, *17*, 1317–1321.

#### Original Ref 43 Turns to 31

- 31. Yang, Z.; Kong, F.; Zhang, M. Groundwater contamination by microcystin from toxic cyanobacteria blooms in Lake Chaohu, China. Environ. *Monit. Assess.* **2016**, *188*, 280.

#### Original Ref 51 Turns to 65

- 65. Bouaïcha, N. Cyanobacterial toxins: Modes of actions, fate in aquatic and soil ecosystems, phytotoxicity and bioaccumulation in agricultural crops. *Chemosphere* **2014**, *96*, 1–15.

#### Original Ref 73 Turns to 79

- 79. Zhang, Y.; Whalen, J.K.; Sauvé, S. Phytotoxicity and bioconcentration of microcystins in agricultural plants: Meta-analysis and risk assessment. *Environ. Pollut.* **2020**, *272*, 115966.

#### Original Ref 76 Turns to 85

- 85. Cao, Q.; Rediske, R.R.; Yao, L.; Xie, L. Effect of microcystins on root growth, oxidative response, and exudation of rice (*Oryza sativa*). *Ecotoxicol. Environ. Saf.* **2018**, *149*, 143–149.

#### Original Ref 78 Turns to 87

- 87. Corbel, S.; Bouaïcha, N.; Nélieu, S.; Mougin, C. Soil irrigation with water and toxic cyanobacterial microcystins accelerates tomato development. *Environ. Chem. Lett.* **2015**, *13*, 447–452.

#### Original Ref 82 Turns to 95

- 95. Lee, S.; Jiang, X.; Manubolu, M.; Riedl, K.; Ludsin, S.A.; Martin, J.F.; Lee, J. Fresh produce and their soils accumulate cyanotoxins from irrigation water: Implications for public health and food security. *Food Res. Int.* **2017**, *102*, 234–245.

#### Original Ref 86 Turns to 108

- 108. Romero-Oliva, C.S.; Contardo-Jara, V.; Pflugmacher, S. Time dependent uptake, bioaccumulation and biotransformation of cell free crude extract microcystins from Lake Amatitlán, Guatemala by Ceratophyllum demersum, Egeria densa and Hydrilla verticillata. *Toxicon* **2015**, *105*, 62–73.

### Removed References (Original Labeled)

- 54. Wu, X.; Xiao, B.; Li, R.; Wang, C.; Huang, J.; Wang, Z. Mechanisms and factors affecting sorption of microcystins onto natural sediments. *Environ. Sci. Technol.* **2011**, *45*, 2641–2647.
- 55. Yuhui, L.; Wang, Y.; Yin, L.; Pu, Y.; Wang, D. Using the nematode Caenorhabditis elegans as a model animal for assessing the toxicity induced by mirocsystin-LR. *J. Environ. Sci.* **2009**, *21*, 395–401.
- 58. Saqrane, S.; Ghazali, I.E.; Oudra, B.; Bouarab, L.; Vasconcelos, V. Effects of cyanobacteria producing microcystins on seed germination and seedling growth of several agricultural plants. *J. Environ. Sci. Health Part B* **2008**, *43*, 443–451.
- 61. Bouaïcha, N.; Miles, C.O.; Beach, D.G.; Labidi, Z.; Djabri, A.; Benayache, N.Y.; Nguyen-Quang, T. Structural diversity, characterization and toxicology of microcystins. *Toxins* **2019**, *11*, 714.
- 75. Sedan, D.; Malaissi, L.; Vaccarini, C.A.; Ventosi, E.; Laguens, M.; Rosso, L.; Giannuzzi, L.; Andrinolo, D. [D-Leu1] MC-LR has lower PP1 inhibitory capability and greater toxic potency than MC-LR in animal and plant tissues. *Toxins* **2020**, *12*, 632.
- 77. Do Carmo Bittencourt-Oliveira, M.; Cordeiro-Araújo, M.K.; Chia, M.A.; de Toledo Arruda-Neto, J.D.; de Oliveira, Ê.T.; dos Santos, F. Lettuce irrigated with contaminated water: Photosynthetic effects, antioxidative response and bioaccumulation of microcystin congeners. *Ecotoxicol. Environ. Saf.* **2016**, *128*, 83–90.
- 79. Pflugmacher, S. Reduction in germination rate and elevation of peroxidase activity in Zea mays seedlings due to exposure to different microcystin analogues and toxic cell free cyanobacterial crude extract. *J. Appl. Bot. Food Qual.* **2007**, *81*, 45–48.
- 83. Cevallos-Casals, B.A.; Cisneros-Zevallos, L. Impact of germination on phenolic content and antioxidant activity of 13 edible seed species. *Food Chem.* **2010**, *119*, 1485–1490.
- 87. Verbitsky, V.B.; Kurbatova, S.A.; Berezina, N.A.; Korneva, L.G.; Meteleva, N.Y.; Makarova, O.S.; Sharov, A.N.; Ershov, I.Y.; Malysheva, O.A.; Russkikh, Y.V.; et al. Responses of aquatic organisms to cyanobacteria and Elodea in microcosms. *Dokl Biol. Sci.* **2019**, *488*, 136–140.
- 89. Zanchett, G.; Oliveira-Filho, E.C. Cyanobacteria and cyanotoxins: From impacts on aquatic ecosystems and human health to anticarcinogenic effects. *Toxins* **2013**, *5*, 1896–1917.
- 90. Saqrane, S.; Ouahid, Y.; El Ghazali, I.; Oudra, B.; Bouarab, L.; del Campo, F.F. Physiological changes in Triticum durum, Zea mays, Pisum sativum and Lens esculenta cultivars, caused by irrigation with water contaminated with microcystins: A laboratory experimental approach. *Toxicon* **2009**, *53*, 786–796.

### Newly Added References (Reordered Labeled)

- 27. Zhang, Y; Husk, B.R.; Dinh, Q.T.; Sanchez, J.S.; Sauvé, S.; Whalen, J.K. Quantitative screening for cyanotoxins in soil and groundwater of agricultural watersheds in Quebec, Canada. *Chemosphere* **2021**, *274*, 129781.
- 32. *Groundwater: Understanding and Protecting Our Hidden Resource*. U.S. Environmental Protection Agency: Washington DC, USA, 2021.
- 34. Pfister, S.; Bayer, P.; Koehler, A.; Hellweg, S. Environmental impacts of water use in global crop production: Hotspots and trade-offs with land use. *Environ. Sci. Technol.* **2011**, *45*, 13.
- 55. Chittora, D.; Meena, M.; Barupal, T.; Swapnil, P. Cyanobacteria as a source of biofertilizers for sustainable agriculture. *Biochem. Biophys. Rep.* **2020**, *22*, 100737.
- 56. Xiang, L.; Li, Y.W.; Wang, Z.R.; Liu, B.L.; Zhao, H.M.; Li, H.; Cai, Q.Y.; Mo, C.H.; Li, Q.X. Bioaccumulation and phytotoxicity and human health risk from microcystin-LR under various treatments: A pot study. *Toxins* **2020**, *12*, 523.
- 58. Sinha, R.; Soni, B.K.; Agarwal, S.M.; Shankar, B.; Hahn, G.E. Vermiculture for organic horticulture: Producing chemical-free, nutritive and health protective foods by earthworms. *Agric. Sci.* **2013**, *1*, 17–44.
- 59. Li, Y.; Wang, Y.; Yin, L.; Pu, Y.; Wang, D. Using the nematode *Caenorhabditis elegans* as a model animal for assessing the toxicity induced by microcystin-LR. *J. Environ. Sci.* **2009**, *21*, 395–401.
- 61. Gurtler, J.B.; Doyle, M.P.; Erickson, M.C.; Jiang, X.; Millner, P.; Sharma, M. Composting to inactivate foodborne pathogens for crop soil application: A review. *J. Food. Prot.* **2018**, *81*, 1821–1837.
- 74. Tsoumalakou, E.; Papadimitriou, T.; Berillis, P.; Kormas, K.A.; Levizou, E. Spray irrigation with microcystins-rich water affects plant performance from the microscopic to the functional level and food safety of spinach (*Spinacia oleracea* L.). *Sci. Total Envirion.* **2021**, *789*, 147948.
- 75. Wang, N.; Wang. C. Effects of microcystin-LR on the tissue growth and physiological responses of the aquatic plant *Iris pseudacorus* L. *Aquat. Toxicol.* **2018**, *200*, 197–205.
- 76. Chen, G.; Li, Q.; Bai, M.; Chen, Y. Nitrogen metabolism in *Acorus calamus* L. leaves induced changes in response to microcystin-LR at environmentally relevant concentrations. *Bull. Environ. Contam. Toxicol.* **2019**, *103*, 280–285.
- 80. Zhao, W.; Fu, P.; Liu, G.; Zhao, P. Difference between emergent aquatic and terrestrial monocotyledonous herbs in relation to the coordination of leaf stomata with vein traits. *AoB PLANTS* **2020**, *12*, plaa047.
- 81. Chen, J.; Song, L.; Dai, J.; Gan, N.; Liu, Z. Effects of microcystins on the growth and the activity of superoxide dismutase and peroxidase of rape (*Brassica napus* L.) and rice (*Oryza sativa* L.). *Toxicon* **2004**, *15*, 393–400.
- 83. Gehringer, M.M.; Kewada, V.; Coates, N.; Downing, T.G. The use of *Lepidium sativum* in a plant bioassay system for the detection of microcystin-LR. *Toxicon* **2003**, *41*, 871–876.
- 84. Pflugmacher, S.; Jung, K.; Lundvall, L.; Neumann, S.; Peuthert, A. Effects of cyanobacterial toxins and cyanobacterial cell-free crude extract on germination of alfalfa (*Medicago sativa*) and induction of oxidative stress. *Environ. Toxicol. Chem.* **2006**, *25*, 381–2387.
- 89. El-Sheekh, M.M.; Khairy, H.M.; El-Shenody, R. Effects of crude extract of *Microcystis aeruginosa* on germination, growth and chlorophyll content of *Zea mays* L. *Bangladesh J. Bot.* **2013**, *42*, 295–300.
- 90. Järvenpää, S.; Lundberg-Niinistö, C.; Sjövall, O.; Tyystjärvi, E.; Meriluoto, J. Effects of microcystins on broccoli and mustard, and analysis of accumulated toxin by liquid chromatography-mass spectrometry. *Toxicon* **2007**, *49*, 865–874.
- 91. Hereman, T.C.; Bittencourt-Olveira, M.C. Bioaccumulation of microcystins in lettuce. *J. Phycol.* **2012**, *48*, 1535–1537.
- 92. Lahrouni, M.; Oufdou, K.; El Khalloufi, F.; Benidire, L.; Albert, S.; Göttfert, M.; Caviedes, M.A.; Rodriguez-Llorente, I.D.; Oudra, B.; Pajuelo, E. Microcystin-tolerant *Rhizobium* protects plants and improves nitrogen assimilation in *Vicia faba* irrigated with microcystin-containing waters. *Environ. Sci. Pollut. Res. Int.* **2016**, *23*, 10037–10049.
- 93. Bittencourt-Olveira, M.C.; Cordeiro-Araújo, M.K.; Chia, M.A.; Arrudo-Neto, J.D.; de Oliveira, E.T.; dos Santos, F. Lettuce irrigated with contaminated water: Photosynthetic effects, antioxidative response and bioaccumulation of microcystin congeners. *Ecotoxicol. Environ. Safi.* **2016**, *128*, 83–90.
- 94. Haida, M.; El Khalloufi, F.; Mugani, R.; Redouane, E.M.; Campos, A.; Vasconcelos, V.; Oudra, B. Effects of irrigation with microcystin-containing water on growth, physiology, and antioxidant defense in strawberry *Fragaria vulgaris* under hydroponic culture. *Toxins* **2022**, *14*, 198.
- 97. Lucini, L.; Bernardo, L. Comparison of proteome response to saline and zinc in lettuce. *Front. Plant Sci.* **2015**, *16*, 240.
- 98. Levizou, E.; Statiris, G.; Papadimitriou, T.; Laspidou, C.S.; Kormas, K.A. Lettuce facing microcystins-rich irrigation water at different developmental stages: Effects on plant performance and microcystins bioaccumulation. *Exotoxicol. Environ. Saf.* **2017**, *143*, 193–200.
- 101. Cao, Q.; Wan, X.; Shu, X.; Xie, L. Bioaccumulation and detoxifixation of microcystin-LR in three submerged macrophytes: The important role of glutathione biosynthesis. *Chemosphere* **2019**, *225*, 935–942.
- 103. Pflugmacher, S.; Wiegand, C.; Beattie, K.A.; Codd, G.A.; Steinberg, C.E.W. Uptake of the cyanobacterial hepatotoxin microcystin-LR by aquatic macrophytes. *Appl. Bot.* **1998**, *72*, 228–232.
- 104. Cao, Q.; Liu, W.; Jiang, W.; Shu, X.; Xie, L. Glutathione biosynthesis plays an important role in microcystin-LR depurination in lettuce and spinach. *Environ. Pollut.* **2019**, *253*, 599–605.
- 105. Yin, L.; Huang, J.; Li, D.; Liu, Y. Microcystin-RR uptake and its effects on the growth of submerged macrophyte *Vallisneria natans* (lour.) hara. *Environ. Toxicol.* **2005**, *20*, 308–313.
- 106. Wang, Z.; Xiao, B.; Song, L.; Wang, C.; Zhang, J. Responses and toxin bioaccumulation in duckweed (*Lemna minor*) under microcystin-LR, linear alkybenzene sulfonate and their joint stress. *J. Hazard Mater.* **2012**, *229–230*, 137–144.
- 107. Song, L.; Chen, W.; Peng, L.; Wan, N.; Gan, N.; Zhang, X. Distribution and bioaccumulation of microcystins in water columns: A systematic investigation into the environmental fate and the risks associated with microcystins in Meilang Bay, Lake Taihu. *Water Res.* **2007**, *41*, 2853–2864.
- 110. Máthé, C.; M-Hamvas, M.; Vasas, G.; Surányi, G.; Bácsi, I.; Beyer, D.; Tóth, S.; Tímár, M.; Borbély, G. Microcystin-LR, a cyanobacterial toxin, induces growth inhibition and histological alterations in common reed (*Phragmites australis*) plants regenerated from embryogenic calli. *New Phytol.* **2007**, *176*, 824–835.
- 111. Xiao, F.G.; Zhao, X.L.; Tang, J.; Gu, X.H.; Zhang, J.P.; Niu, W.M. Necessity of screening water chestnuts for microcystins after cyanobacterial blooms break out. *Arch. Environ. Contam. Toxicol.* **2009**, *57*, 256–263.

The authors sincerely apologize for the errors in the original publication. The changes do not affect the original meaning of the work. This correction was approved by the Academic Editor. The original publication has also been updated.
